# HECTD3 Mediates an HSP90-Dependent Degradation Pathway for Protein Kinase Clients

**DOI:** 10.1016/j.celrep.2017.05.078

**Published:** 2017-06-20

**Authors:** Zhaobo Li, Lihong Zhou, Chrisostomos Prodromou, Velibor Savic, Laurence H. Pearl

**Affiliations:** 1 Genome Damage and Stability Centre, School of Life Sciences, University of Sussex, Falmer, Brighton BN1 9QR, UK; 2 Brighton and Sussex Medical School, University of Sussex, Falmer, Brighton BN1 9PX, UK

**Keywords:** chaperone, ubiquitin, inhibitor, ATPase

## Abstract

Inhibition of the ATPase cycle of the HSP90 chaperone promotes ubiquitylation and proteasomal degradation of its client proteins, which include many oncogenic protein kinases. This provides the rationale for HSP90 inhibitors as cancer therapeutics. However, the mechanism by which HSP90 ATPase inhibition triggers ubiquitylation is not understood, and the E3 ubiquitin ligases involved are largely unknown. Using a siRNA screen, we have identified components of two independent degradation pathways for the HSP90 client kinase CRAF. The first requires CUL5, Elongin B, and Elongin C, while the second requires the E3 ligase HECTD3, which is also involved in the degradation of MASTL and LKB1. HECTD3 associates with HSP90 and CRAF in cells via its N-terminal DOC domain, which is mutationally disrupted in tumor cells with activated MAP kinase signaling. Our data implicate HECTD3 as a tumor suppressor modulating the activity of this important oncogenic signaling pathway.

## Highlights

• siRNA screen identifies factors regulating HSP90-directed client degradation
• HECTD3 promotes CRAF degradation after HSP90 ATPase inhibition
• HECTD3 interacts with HSP90-CDC37-CRAF via its DOC domain
• CRAF-dependent tumor cells downregulate HECTD3 E3 ligase activity

## Graphical Abstract

**Figure:**
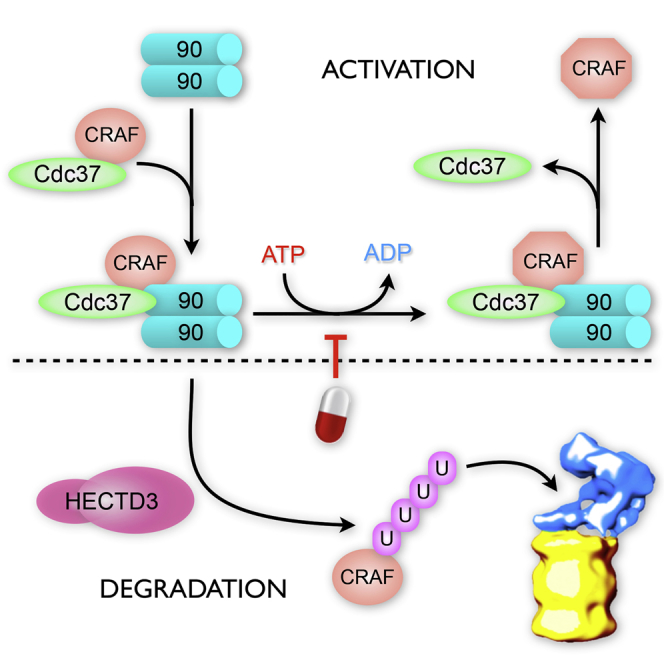

## Teaser

Li et al. develop a cellular assay for HSP90-inhibitor-triggered degradation of protein kinase clients and screen for components of the ubiquitin/proteasome system that mediate this. They identify HECTD3 as an E3 ligase that directs CRAF degradation and demonstrate its interaction with HSP90/kinase complexes via its DOC domain.

## Introduction

The HSP90 molecular chaperone is responsible for the stabilization and biological activity of a diverse set of “clients,” including clinically important proteins such as nuclear hormone receptors and a broad range of protein kinases (Taipale et al., 2010). The involvement of the HSP90 (heat shock protein 90) system in the cellular stabilization of oncogenic protein kinases such as ErbB2, BRaf-V600E, FGFR-G719S, BCR-ABL, and EML4-ALK has marked it as a prime target for drug discovery, and a number of potent HSP90 inhibitors are at various stages of clinical trial in a range of tumor types (Neckers and Workman, 2012). These compounds act as competitive inhibitors of ATP binding to the N-terminal domain of the chaperone molecule, blocking the ATPase-coupled conformational cycle that is essential for HSP90s activity (Ali et al., 2006, Panaretou et al., 1998, Prodromou et al., 2000).

Early studies showed that client proteins such as CRAF and ErbB2 become ubiquitylated and degraded by the proteasome in cells treated with the natural-product HSP90 inhibitor geldanamycin (Chavany et al., 1996, Schulte et al., 1995), even before its biochemical mode of action as an ATP-competitive inhibitor was revealed (Prodromou et al., 1997, Roe et al., 1999). This phenomenon has been robustly repeated for many HSP90-dependent protein kinases using a range of different inhibitor chemotypes (Banerji et al., 2005, Chiosis et al., 2001, Sharp and Workman, 2006, Sharp et al., 2007) and is widely accepted as the hallmark of an HSP90 client protein. Protein kinase clients of HSP90 are also ubiquitylated and degraded when their interaction with the HSP90 co-chaperone CDC37, and consequent recruitment to the HSP90 machinery, is blocked by ATP-competitive protein kinase inhibitors (Polier et al., 2013). Whether this proceeds through the same pathway as the HSP90-inhibitor-triggered degradation is uncertain.

Ubiquitylation involves a cascade of enzymatic reactions, starting with the ATP-dependent activation of ubiquitin by the E1-activating enzyme and its covalent attachment to an E2-conjugating enzyme via a thioester bond connecting the α-carboxyl at the C terminus of ubiquitin and a cysteine side chain of the E2. Transfer of ubiquitin from the E2-ubiquitin (E2-Ub) conjugate to the target protein is catalyzed by an E3 ubiquitin ligase enzyme. E3 enzymes provide the target specificity of the ubiquitylation process and encapsulate the ability to recognize a specific feature of the target protein—the degron—that marks it for modification.

While a number of E3 ligases, such as CHIP and cullin-RING ubiquitin ligases (CRLs) based upon CUL5, have been implicated, there is no consensus on the pathway by which HSP90-dependent client proteins become ubiquitylated and targeted for degradation. In particular, there is no understanding of the nature of the degron presented by the target protein in the context of a complex with HSP90 in which the chaperone ATPase cycle is inhibited or when the target protein is deprived of chaperone interaction by an ATP-competitive kinase inhibitor.

To gain further insight into these questions, we have developed a cell-based HSP90 client protein degradation assay that is amenable to high-throughput screening, and we have performed a focused siRNA (small interfering RNA) screen of components of the cellular ubiquitylation system, in order to identify the factors involved. Our data confirm a role for CUL5-based systems but identify a major new route for HSP90 client degradation via a member of the HECT-domain family of E3 ligases.

## Results

Client protein degradation following pharmacological inhibition of HSP90 can be followed by immunoblots of cell lysates. However, this format is only suitable for determining the involvement of a limited number of candidate genes. Therefore, we set out to develop an assay format in which degradation of a client protein could be monitored in a highly parallel fashion suitable for use in a broad siRNA screen of the components of the ubiquitin-proteasome system.

### A Fluorescence-Based Assay for Client Protein Degradation

**Figure 1:**
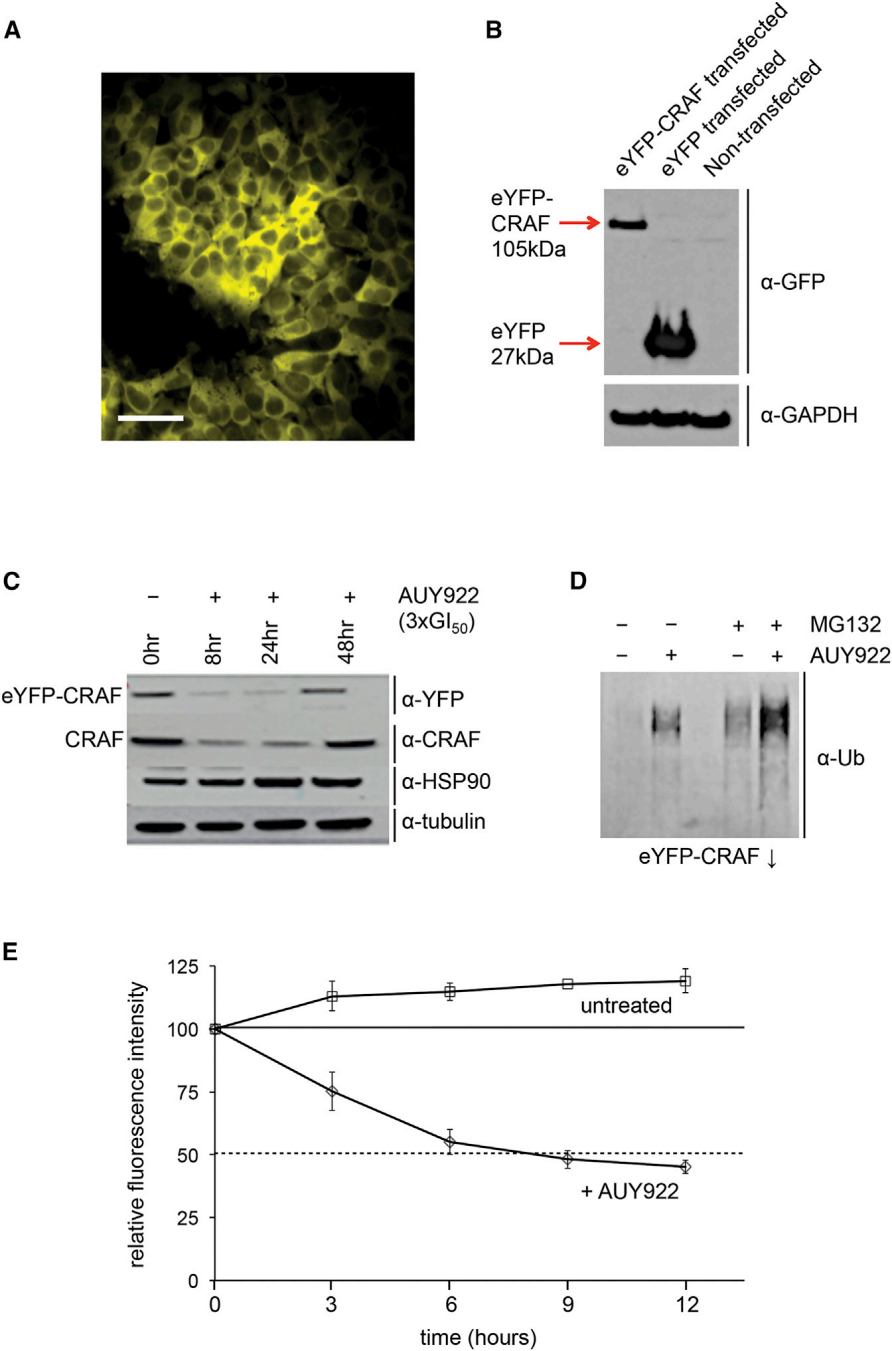
HSP90-Dependent Degradation of eYFP-CRAF Fusion Protein (A) eYFP-CRAF stably transfected into HEK293 retains the cytoplasmic distribution displayed by endogenous CRAF. Scale bar, 50 μm. (B) eYFP-CRAF transfected into HEK293 expresses a stable full-length protein with no detectable free eYFP, so that all cellular fluorescence can be attributed to levels of the fusion protein. (C) eYFP-CRAF, like endogenous CRAF, is degraded in HEK293 cells following treatment with the HSP90 ATPase inhibitor AUY922. (D) Treatment of transfected cells with AUY922 promotes ubiquitylation of eYFP-CRAF, as visualized in an anti-ubiquitin western blot. The levels of ubiqutylated protein detected are enhanced by the proteasome inhibitor MG132. These data confirm that eYFP-CRAF is degraded in the same way as previously shown for endogenous CRAF. (E) Treatment of transfected cells with AUY922 over 8 hr gives an ∼50% reduction in relative fluorescence intensity of transfected cells compared with untreated cells. Error bars show SD.

The proto-oncogene kinases BRAF and CRAF are well-documented HSP90 client proteins that have previously been shown to be ubiquitylated and degraded in tumor cell lines treated with the HSP90 inhibitor AUY922 (Sharp et al., 2007). However, AUY922, like other HSP90 inhibitors, strongly inhibits cell growth and also promotes apoptosis in tumor cell lines such as HT29 and HCT116, which are addicted to mitogen-activated protein kinase (MAPK) signaling mediated by RAF kinases, potentially confounding reliable measurement of protein levels. Furthermore, tumor cells are likely to have highly perturbed protein degradation pathways that reflect their idiosyncratic growth requirements. Therefore, we explored a number of alternatives and settled on HEK293 cells, which are virally immortalized non-cancer cells not known to be dependent on MAPK signaling for survival and growth. For facile measurement of client protein levels, we explored a number of reporter constructs in which a fluorescent protein was fused to BRAF, CRAF, or their isolated kinase domains. We found that full-length CRAF with an N-terminal enhanced yellow fluorescent protein (eYFP) fusion (Experimental Procedures) could be stably expressed at visible levels in HEK293 cells (Figures 1A and 1B) and displayed a sub-cellular distribution similar to that of endogenous CRAF in the absence of oncogenic RAS (Marais et al., 1997). Expression of the eYFP-CRAF fusion had no effect on the concentration of AUY922 that gave 50% growth inhibition (GI_50_) in the HEK293 cells, indicating that the expression of the eYFP-CRAF fusion was neither toxic nor mitogenic (Figure S1A). We also determined a concentration of AUY922 (3 × GI_50_) that, while substantially decreasing cell growth relative to untreated cells, did not cause any decrease in total cell count over a 72-hr incubation period. This concentration also had virtually no effect on cell viability after 12 hr; therefore, we settled on this concentration (3 × GI_50_) in all subsequent assays (Figures S1B and S1C). We observed a substantial decrease compared to control in levels of both endogenous CRAF and the eYFP-CRAF fusion protein at time points between 8 and 24 hr after treatment of transfected cells with AUY922 at this concentration (Figure 1C). Consistent with this, eYFP-CRAF protein immunoprecipitated from cells treated with AUY922 cross-reacted with an anti-ubiquitin antibody, and this was substantially enhanced by the addition of the proteasome inhibitor MG132 (Figure 1D). Taken together these data confirm that the addition of the N-terminal eYFP did not interfere with the well-described HSP90-associated and ubiquitin-dependent degradation of CRAF following HSP90 inhibition. Finally, we measured the effect of treatment with AUY922 at our standardized dose on the fluorescence signal intensity of the eYFP-CRAF stably transfected cells, and we found a reproducible ∼50% decrease in intensity over 8 hr relative to untreated cells (Figure 1E). This was consistent with the loss of signal in the western blots and sufficient to provide a robust and quantitative measure of drug-triggered protein degradation in viable cells that is amenable to automated screening.

### siRNA Screen for Mediators of CRAF Degradation

The HEK293 cells stably expressing eYFP-CRAF were screened against a siRNA library (Dharmacon) directed against all human E1 ubiquitin-activating enzymes, E2 ubiquitin-conjugating enzymes, and components of CRLs and HECT E3 ubiquitin ligase systems, among others. Degradation of eYFP-CRAF was initiated by the addition of AUY922, and experimental values were determined by measuring the change in fluorescence intensity in the treated cells over 8 hr (see Experimental Procedures).

**Figure 2:**
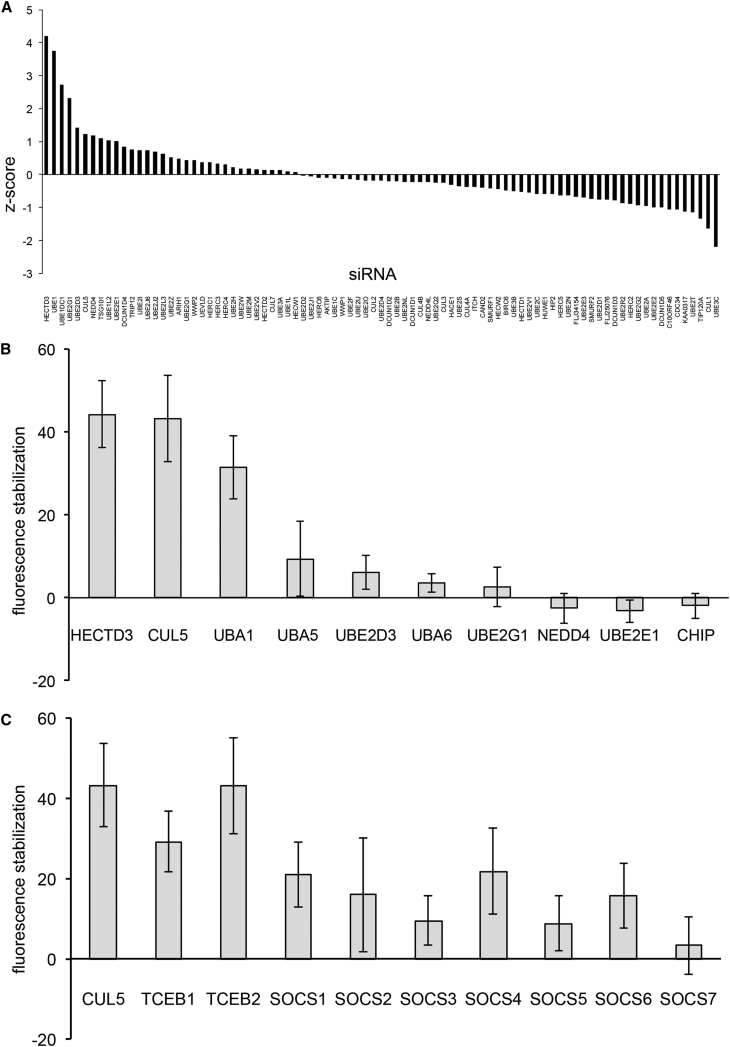
siRNA Screen for Factors Mediating eYFP-CRAF Degradation (A) Waterfall plot of siRNA-targeted genes (horizontal) versus the stabilization of fluorescence in cells expressing eYFP-CRAF transfected with test siRNAs and treated with AUY922 relative to treated cells transfected with a control siRNA. (B) Repeat siRNA knockdowns of “hits” identified in (A); knockdown of HECTD3, CUL5, and UBA1 (also known as UBE1) using different siRNAs from those in (A) gave robust and reproducible stabilization of eYFP-CRAF on AUY922 treatment. Measurements are averages of four (UBA1 only) or six replicates, and error bars indicate SD. Stabilization relative to control following knockdown of HECTD3, CUL5, and UBA1 is significant in a paired t test (p < 0.0001 for HECTD3 and CUL5, and p = 0.001 for UBA1). (C) Same as in (B), but comparing the siRNA knockdown of CUL5 (same data as in B) with the knockdown of known CUL5 CRL complex components, relative to siRNA control. Knockdown of the accessory scaffold components TCEB1 (also known as Elongin C) and TCEB2 (also known as Elongin B) stabilized eYFP-CRAF to a degree comparable to that of CUL5 (p = 0.00015 for TCEB1, and p = 0.00017 for TCEB2), consistent with their essential involvement in CUL5-based CRL E3 ligase complexes. However, none of the SOCS proteins gave comparable stabilization (all significant measurements at least p < 0.01). Values are averages of six replicates and error bars show SD.

Genes were ranked by the relative stabilization of fluorescence following AUY922 treatment when expression of the encoded protein was knocked down, compared to the level following AUY922 treatment in the presence of a non-targeting control siRNA (see Experimental Procedures) (Figure 2A; Table S1). Of the 87 genes tested, 10 stabilized eYFP-CRAF by 15% or greater, compared with the control. Among these were the E1 ubiquitin-activating enzymes UBE1 (also known as UBA1) and UBE1L2, as well as the ubiquitin-like protein E1 enzyme UBE1DC1. As E1 enzymes are required for all ubiquitylation processes, the identification of UBE1 as a factor making a substantial contribution to CRAF degradation provides a critically important positive control that validates the screen. Three E2 ubiquitin-conjugating enzymes also feature among the highest ranked genes: UBE2D3 (also known as UbcH5C), which can act as an initiator of ubiquitin chains and in K11 and K48-specific chain elongation (UniProt: P61077); UBE2G1 (also known as Ubc7), which catalyzes K48 or K63 chain elongation (UniProt: P62253); and UBE2E1 (UbcH6), which catalyzes K48 chain elongation (UniProt: P51965). Components of four E3 ligase systems also featured in the ten highest ranked genes. These include CUL5, the core scaffold of a large group of CRLs (Lydeard et al., 2013); TSG101, a ubiquitin-binding component of the ESCRT1 system (Zhang et al., 2014); and two HECT-domain E3 ligases—NEDD4, implicated in the regulation of a range of membrane-associated signaling proteins and ion channels (Zou et al., 2015), and HECTD3, whose biology is, as yet, poorly defined.

### Involvement of CUL5 and HECTD3

To verify the involvement of these genes in mediating HSP90-associated and ubiquitin-dependent degradation of eYFP-CRAF, we repeated the fluorescence stabilization assays with individually designed siRNAs distinct from the pools used in the screens. None of the E2s in the top-ten hits from the original screen gave stabilization of eYFP-CRAF >10% when knocked down in the repeat experiments, nor did the repeat siRNA knockdown of the E3 NEDD4. However, repeat siRNA knockdown of CUL5 or HECTD3 caused robust, repeatable, and statistically highly significant (p < 0.0001 in paired t test) stabilization of eYFP-CRAF fluorescence at levels comparable to that obtained with repeat siRNA knockdown of UBE1 (Figure 2B). Knockdown of the HSP90/HSP70-associated U-box E3 ligase CHIP/STUB1, which has previously been implicated in HSP90-inhibitor-triggered degradation of ErbB2 (Xu et al., 2002, Zhou et al., 2003) and a range of other ubiquitylation events (Edkins, 2015), had no significant effect (p > 0.1 in paired t test) on the stability of eYFP-CRAF in this system.

HECTD3, which gave the strongest signal in the initial screen, belongs to a class of E3 ubiquitin ligases in which substrate recognition, E2 recruitment, and catalytic activity are often encapsulated in a single polypeptide chain (Rotin and Kumar, 2009). CUL5, however, is the common core “scaffold” component of a family of multiprotein complexes, the Elongin BC-CUL2/5-SOCS-box (ECS) E3 ubiquitin ligases, where it provides the binding sites for a catalytic RING finger protein (RBX1 or RBX2) required for Ubq-E2 recruitment and for the TCEB2-TCEB1 (also known as Elongin B-Elongin C) heterodimer. This latter mediates recruitment of one of seven SOCS-box-containing proteins that provide specificity for individual ubiquitination substrates of this E3 system (Lydeard et al., 2013). Consistent with the involvement of CUL5 in eYFP-CRAF degradation, but in contradiction to earlier studies in tumor cell lines that implicated CUL5 in HSP90 client protein degradation (Ehrlich et al., 2009, Samant et al., 2014) independently of TCEB1/2, we found that siRNA knockdown of the CUL5 partner scaffold proteins TCEB2 and, to a lesser degree, TCEB1 also elicited substantial stabilization of eYFP-CRAF fluorescence. However, none of the seven SOCS-box proteins gave a comparable signal to knockdown of the TCEB1/2 proteins that recruit them, although siRNA knockdown of both SOCS1 and SOCS4 stabilized eYFP-CRAF fluorescence by ∼20% (Figure 2C). These data suggest that eYFP-CRAF degradation by this system is mediated by a conventional CUL5-TCEB1/2 core, but with target selectivity either provided redundantly by multiple SOCS-box proteins or by as-yet-unidentified proteins that are recruited via the TCEB1/2 (Elongin B/C) adaptor scaffold.

### Client Protein Specificity of HECTD3

**Figure 3:**
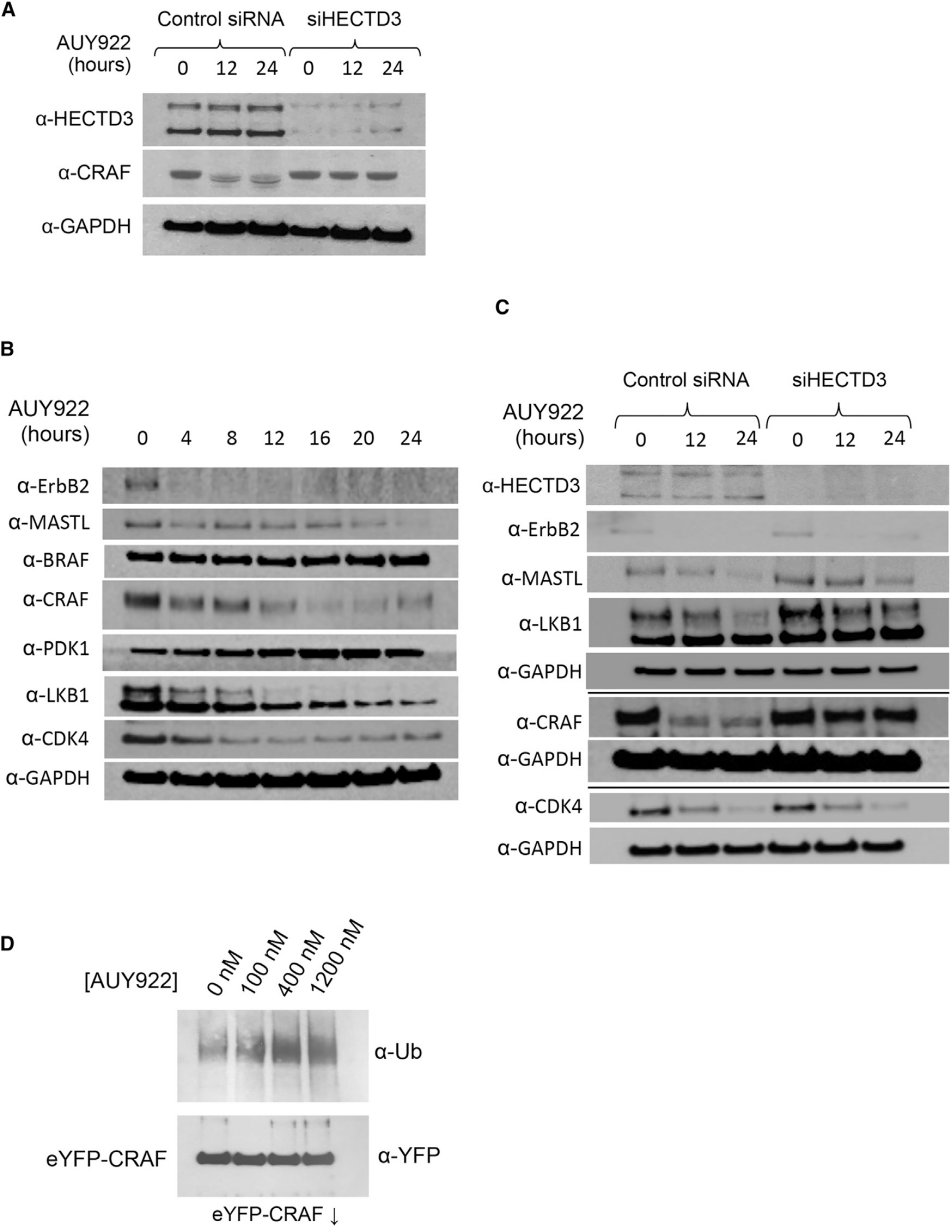
Kinase Specificity of HECTD3 (A) Western blot of endogenous CRAF in HEK293 cell lysates following 24-hr treatment with AUY922. Substantial degradation is observed in cells transfected with control siRNA, but CRAF levels are stabilized in cells transfected with siRNA against HECTD3. Both immunoreactive bands to the HECTD3 antibody are knocked down by the siRNA; see below. GAPDH levels provide a loading control. (B) Western blots of known HSP90 protein kinase clients in HEK293 lysates and their response to treatment of cells with AUY922. Levels of ErbB2, MASTL, CRAF, LKB1, and CDK4, but not BRAF or PDK1, decrease in response to HSP90 inhibition. (C) MASTL, LKB1, and CRAF degradation following AUY922 treatment is reduced in HEK293 cells transfected with siRNA to HECTD3 compared to a control siRNA. Knockdown of HECTD3 had no clear effect on AUY922-triggered degradation of ErbB2 or CDK4. The panel is a montage of three separate experiments; GAPDH is a loading control for each. (D) Western blot of eYFP-CRAF immunoprecipitated from cell lysates of HEK293 cells treated with increasing doses of AUY922 post-lysis. Ubiquitylation of eYFP-CRAF can be enhanced in HEK293 cell lysates, as well as in intact cells, by the addition of AUY922 in a dose-dependent fashion.

Although the eYFP N-terminal fusion did not affect the susceptibility of CRAF to HSP90-associated degradation, we wanted to eliminate the possibility that the poorly characterized HECTD3 E3 ligase identified by the screen, and subsequently confirmed in individual experiments, reflects an idiosyncratic feature of the eYFP fusion protein rather than specificity for the CRAF kinase itself. By western blot, we observed robust degradation of endogenous native CRAF in untransformed HEK293 cells treated with control siRNA 24 hr after the addition of AUY922, but this was substantially reduced in cells in which HECTD3 was knocked down, confirming that endogenous CRAF is a bona fide degradation target of HECTD3 (Figure 3A).

We also wanted to determine whether CRAF was the only HSP90 protein kinase client whose degradation was mediated by HECTD3. We immunoblotted HEK293 cells for a range of documented HSP90 protein kinase clients in addition to CRAF and found that ErbB2, BRAF, MASTL, LKB1, PDK1, and CDK4 were all expressed at detectable levels in HEK293 cells. Of these, we observed robust degradation of ErbB2, MASTL, LKB1, and CDK4 when cells were treated with AUY922 at the same exposure used for CRAF, albeit with different kinetics for the individual proteins. PDK1 and BRAF, which is wild-type in HEK293 cells, were not noticeably degraded over a 24-hr period under the same conditions (Figure 3B). siRNA knockdown of HECTD3 diminished HSP90-inhibitor-triggered degradation of LKB1 and MASTL, albeit to a lesser degree than CRAF, but had little effect on degradation of CDK4 and ErbB2 (Figure 3C). Interestingly, drug-induced ubiquitylation of CRAF did not require intact cells and could be observed in lysates from HEK293 cells expressing eYFP-CRAF on the addition of AUY922 in a dose-dependent fashion (Figure 3D).

### Kinase-Inhibitor-Dependent Degradation

We previously showed that ATP-competitive kinase inhibitors block the association of a range of protein kinase clients with the kinase-specific co-chaperone CDC37 and thereby deprive them of access to the HSP90 chaperone system, resulting in their ubiquitylation and degradation (Polier et al., 2013). Whether this HSP90-independent pathway of client degradation operates through the same mechanism as the HSP90-dependent pathway is currently unknown.

Treatment of HEK293 cells with sorafenib substantially inhibited MAPK pathway signaling and promoted some degradation of eYFP-CRAF over 24 hr, although to a much smaller degree than in our previous observations for (1) vemurafenib and BRAFV600E, (2) lapatinib and ErbB2, and (3) erlotinib and EGFRG719S (Polier et al., 2013) in tumor cells (Figure S2A). Knockdown of HECTD3 in these cells had little effect on this response to sorafenib, compared with treatment with a control siRNA, suggesting that HECTD3 probably does not play a major role in the kinase degradation pathway triggered by chaperone deprivation due to kinase inhibitor blockade of CDC37 binding.

Finally, we sought to determine whether HECTD3 is involved in general CRAF turnover in unstressed conditions where there is full access to a functional CDC37/HSP90 chaperone system. HEK293 cells expressing eYFP-CRAF were pulse labeled with the methionine mimetic azidohomoalanine and lysed at different times post-labeling (see Experimental Procedures). Labeled protein was tagged with biotin using a CLICK reaction, and eYFP-CRAF was immunoprecipitated and visualized in an anti-biotin western blot (Figure S2B). We observed little difference in the progressive decrease in detectable levels of biotin-labeled eYFP-CRAF over time between cells treated with a control siRNA or with siRNA directed against HECTD3. This shows that, while HECTD3 is a significant player in CRAF degradation in the context of HSP90, it does not play a major role in overall CRAF proteostasis in unstressed conditions.

### HECTD3, HSP90, and CRAF Interaction

**Figure 4:**
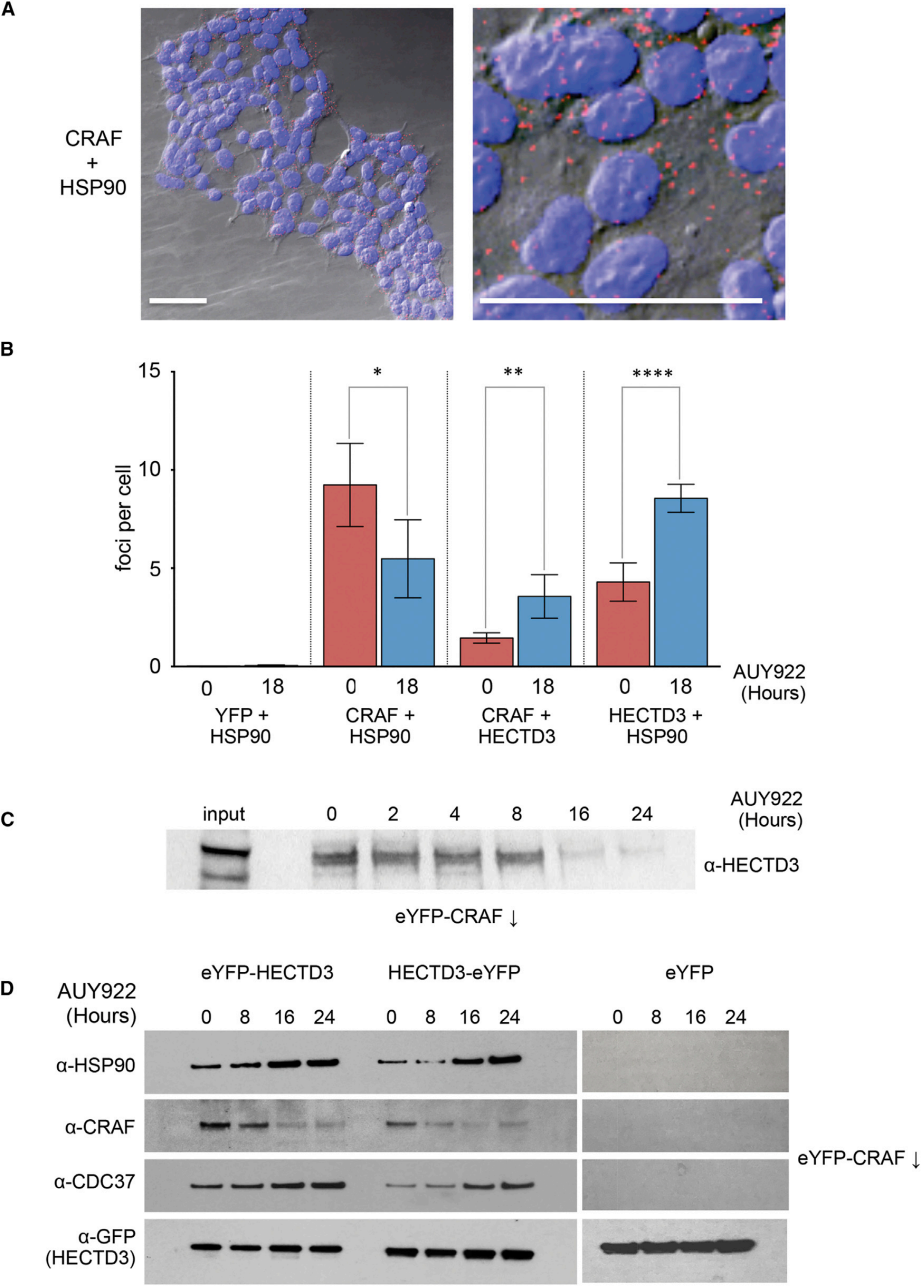
Cellular Association of HSP90, CRAF, and HECTD3 (A) Combined differential interference contrast (Nomarski)/fluorescence image of field of untreated HEK293 cells. Cell nuclei are indicated in blue (DAPI), and co-localized HSP90 and CRAF are visualized as red foci generated by a rolling circle amplification PLA (see Experimental Procedures). Scale bars, 50 μm. Right panel shows a 5× digital zoom of the left panel. (B) Histogram of average PLA foci per cell from analysis of cells before and after treatment with AUY922 for the protein pairs indicated (Figure S3). Proximity of eYFP and endogenous HSP90 was measured in cells expressing eYFP and provides a control for the non-specific signal; all other measurements were in un-transfected HEK293 cells. Values are averages of eight fields and error bars show SD; ^∗^p > 0.05; ^∗∗^p ≤ 0.01; ^∗∗∗∗^p ≤ 0.0001. (C) Western blot of immunoprecipitated eYFP-CRAF from HEK293 cells showing co-immunoprecipitation of HECTD3. The levels of recovered HECTD3 diminish as eYFP-CRAF is degraded following treatment with AUY922. (D) Western blots of HSP90, CRAF, and CDC37 co-immunprecipitated from HEK293 cells expressing eYFP-HECTD3, HECTD3-eYFP, or eYFP, with GFP-trap (see Experimental Procedures). Levels of HSP90 and CDC37 co-immunoprecipitated with the HECTD3 constructs increase with length of drug exposure. No HSP90 CRAF or CDC37 was co-immunoprecipitated from cells expressing eYFP only. The levels of recovered CRAF diminish as it is degraded following treatment with AUY922. GFP immunoreactivity provides a common loading control. See also Figure S3.

Our siRNA data strongly implicate HECTD3 as a specific component of the HSP90-associated, ubiquitin-dependent proteasomal degradation pathway for CRAF in HEK293 cells. However, they do not define whether HECTD3 is involved directly in the recognition and ubiquitylation of CRAF in the context of inhibited HSP90 or whether it is a downstream factor whose influence is indirect. To gain some insight into this, we established a cellular proximity ligation assay (Duolink, Sigma-Aldrich; see Experimental Procedures), which generates a fluorescent focus when two target proteins are within 30–40 nm of each other within the cell, and we used this to determine whether HECTD3 co-localizes with HSP90 and/or CRAF. As a negative control, we looked at the proximity of endogenous HSP90 with overexpressed eYFP in HEK293 cells. As these two abundant proteins are not expected to interact, this provides a control for the background noise of the proximity ligation assay (PLA) system, and very few foci were detectable in untreated cells or cells treated with AUY922 for 18 hr (Figure S3). In contrast, the known interacting proteins HSP90 and CRAF gave a strong proximity signal (Figure 4A), which dropped by nearly half after 18-hr treatment with AUY922, reflecting the depletion of CRAF we observed in treated cells at that time point. A proximity signal was also observed between CRAF and HECTD3 in untreated cells, but this more than doubled in cells treated for 18 hr with AUY922, indicating a large increase in proximity between HECTD3 and CRAF following HSP90 inhibition. This increase in co-localized HECTD3 and CRAF is all the more significant, given the substantial decrease in total cellular CRAF that treatment with the HSP90 inhibitor elicits (Figure 3). A comparably significant increase in proximity between HECTD3 and HSP90 was also observed following drug treatment (Figure 4B). HECTD3 could also be detected in western blots following immunoprecipitation of eYFP-CRAF from treated cells, with the signal decreasing, as eYFP-CRAF is progressively degraded (Figure 4C). To confirm the HECTD3-CRAF interaction, we constructed stable HEK293 lines expressing either a HECTD3-eYFP or an eYFP-HECTD3 fusion protein, or eYFP alone, and used these to co-immunoprecipitate associated proteins. We observed robust specific co-immunoprecipitation of HSP90, the kinase-specific co-chaperone CDC37, and endogenous CRAF with both of the HECTD3 constructs, but not with eYFP alone (Figure 4D). Consistent with the HSP90-inhibitor-dependent ubiquitylation and degradation of CRAF, the levels of HSP90 and CDC37 co-immunoprecipitated with HECTD3 increased with increasing exposure of the cells to AUY922, while the levels of CRAF recovered decreased in line with its progressive degradation. Taken together, these data show that HECTD3 is brought into close physical proximity in cells with both HSP90 and CRAF, as part of the HSP90-inhibitor-induced ubiquitylation and degradation of CRAF, and is most likely involved in a physical complex with both proteins. The persistence of CDC37 in association with HECTD3 and HSP90 following drug treatment is in contrast with previous observations of CUL5, whose presence in kinase immunoprecipitates involving HSP90 was found to coincide with the loss of CDC37 (Samant et al., 2014).

### HECTD3 DOC Domain Mediates CRAF/HSP90 Interaction

**Figure 5:**
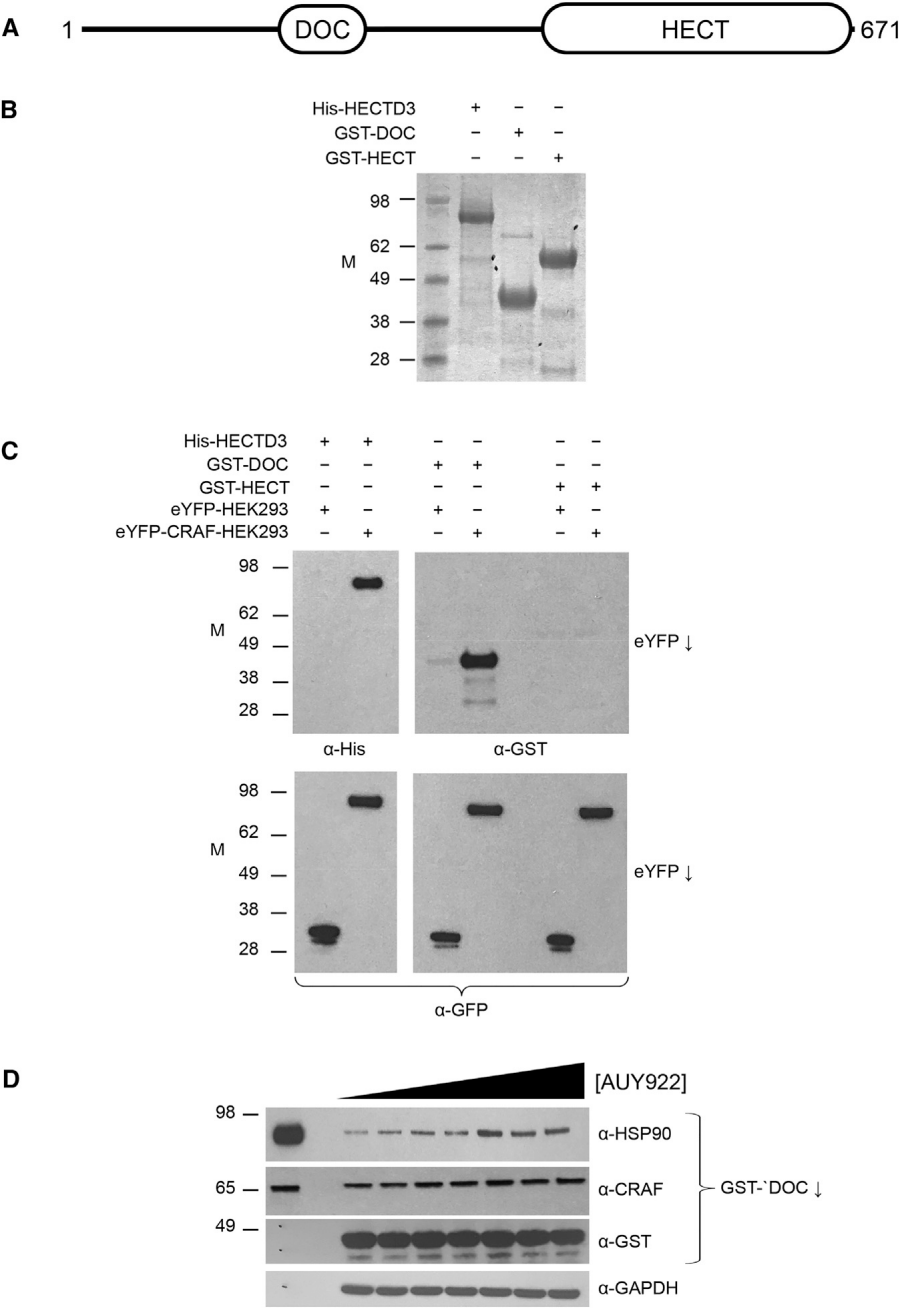
The DOC Domain of HECTD3 Mediates Substrate Interaction (A) Schematic of the domain architecture of HECTD3. Only two defined domains can be recognized: the C-terminal catalytic HECT domain common to all members of the HECT E3 ubiquitin ligase family and a DOC domain homologous to APC10, which occurs midway through the N-terminal region. (B) Coomassie-stained SDS-PAGE of purified recombinant HECTD3 constructs: His_6_-tagged full-length HECTD3 and GST fusions of the isolated DOC and HECT domains. M, molecular weight, in kilodaltons. (C) Top: lysates from HEK293 cells expressing either eYFP or eYFP-CRAF were incubated with purified HECTD3 constructs as in (A), subjected to immunoprecipitation using GFP-Trap (Experimental Procedures), and analyzed by western blot using α-His (left) or α-GST (right). Full-length HECTD3 and the isolated DOC domain, but not the isolated HECT domain, were co-immunoprecipitated from eYPF-CRAF cells. Bottom: loading control for above, western blotted with α-GFP. (D) Purified GST-DOC was added to HEK293 lysates treated with increasing concentrations of the HSP90 inhibitor AUY922 from 0, 100, 200, 400, 800, 1,600, and 3,200 nM and immunoprecipitated. Endogenous CRAF and HSP90 were co-immunoprecipitated, with increased yields at the higher drug concentrations.

HECTD3 has a unique architecture among the HECT-domain E3 ubiquitin ligases (Rotin and Kumar, 2009). Apart from the highly conserved catalytic HECT domain (Marín, 2010) at the C terminus, the only identifiable feature in the HECTD3 amino acid sequence is a DOC domain in the N-terminal region of the protein (Figure 5A). DOC domains also occur in the APC10 subunit of the anaphase-promoting complex (da Fonseca et al., 2011) and in the atypical cullin proteins CUL7 and CUL9 (Dias et al., 2002).

To define which regions of HECTD3 are required for interaction with its CRAF ubiquitylation substrate, we developed recombinant expression systems for full-length HECTD3 and for constructs of the isolated DOC and HECT domains (see Experimental Procedures) (Figure 5B). Full-length His_6_-HECTD3, added to lysates from HEK293 cells expressing the eYFP-CRAF fusion protein, was robustly co-immunoprecipitated by anti-eYFP antibodies, whereas no His_6_-HECTD3 was co-immunoprecipitated when added to cells only expressing eYFP (Figure 5C). A GST (glutathione S-transferase) fusion of the isolated HECTD3-DOC (GST-DOC) domain could also be co-immunoprecipitated from HEK293 cells expressing eYFP-CRAF, whereas a GST fusion of the isolated HECTD3-HECT (GST-HECT) domain was not. In the reverse experiment, GST-DOC added to a HEK293 cell lysate was able to co-precipitate endogenous CRAF and associated HSP90 (Figure 5D). These data strongly implicate the DOC domain as a key determinant of the interaction of HECTD3 with CRAF and HSP90.

### HECTD3 Isoforms and Cell Line Variability

**Figure 6:**
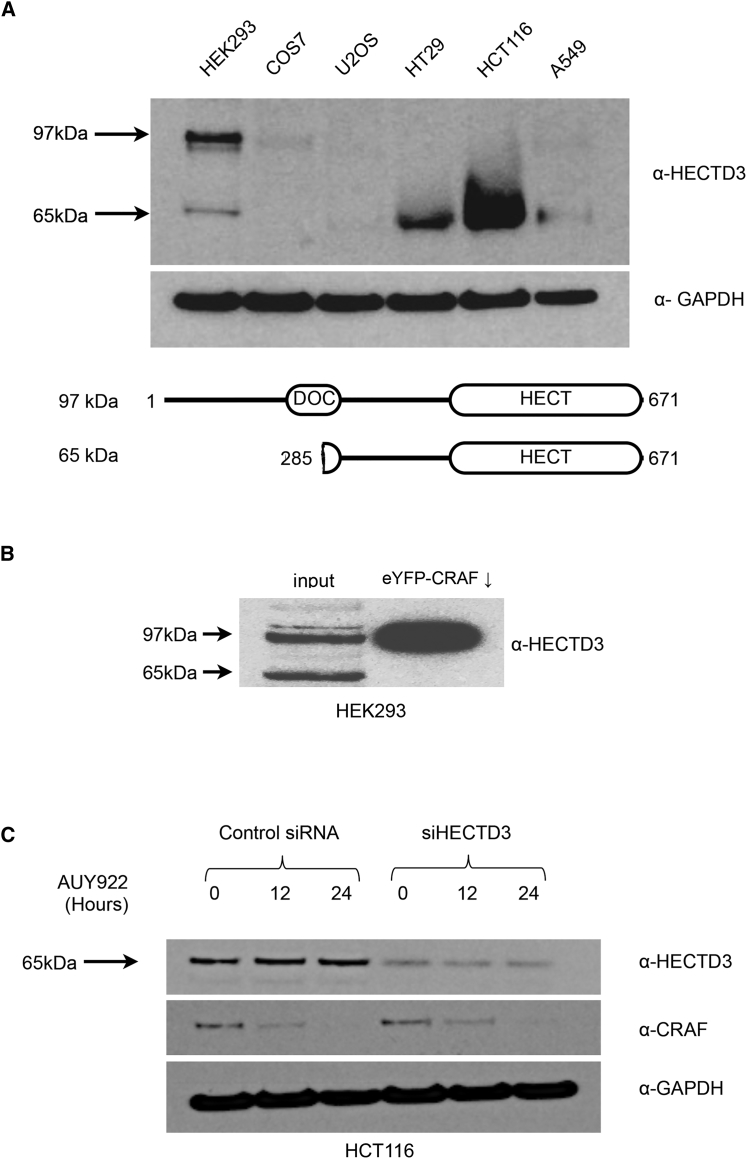
HECTD3 Is Downregulated in Cancer Cell Lines with Activated MAPK Signaling (A) Western blot of HECTD3 in lysates from HEK293, COS7, and four human cancer cells lines: U2OS, HT29, HCT116, and A549. Tumor cell lines either lack immunoreactive protein or express a truncated isoform (also visible in HEK293) that is recognized by the C-terminal epitope of the α-HECTD3 antiserum. The molecular weight of the truncated product corresponds to that predicted for an experimentally documented, alternatively spliced isoform of HECTD3. (B) Western blot of HECTD3 from lysates of HEK293 cells expressing eYFP-CRAF. While both 97-kDa and 65-kDa HECTD3 isoforms are present in the input, only the 97-kDa species corresponding to the full-length protein is co-immunprecipitated with eYFP-CRAF. (C) The truncated splice isoform of HECTD3 in HCT116 cells is effectively knocked down by siRNA, but unlike knockdown of the full-length protein in HEK293 cells, this does not stabilize endogenous CRAF protein to degradation triggered by AUY922. This shows that the 65-kDa isoform is not an active participant in CRAF ubiquitylation and degradation.

Unlike HEK293 cells, tumor cell lines such as HCT116 and HT29, in which HSP90-inhibitor-triggered client protein degradation has been previously studied, are dependent on intense signaling through the MAPK cascade. This, in turn, is critically dependent on the activity of the HSP90 clients CRAF and/or BRAF-V600E (Ehrlich et al., 2009, Samant et al., 2014). Consequently, it is likely that such tumor cells will have adapted to decrease the influence of HSP90-linked degradative pathways for these proteins. Therefore, we looked at the expression of HECTD3 in a range of cell lines. Using a commercial HECTD3 antibody (ab173122, Abcam) that recognizes an epitope close to the C terminus of the protein, we performed western blots of cell extracts from HEK293, COS7, U2OS, HT29, HCT116, and A549 cells (Figure 6A). Immunoreactive bands for proteins with molecular weights 65 kDa and/or 97 kDa were visible, but the intensity of these differed significantly between cell lines. The 97-kDa band, consistent with the predicted molecular weight of the full-length protein encoded by the *HECTD3* gene (97,113 Da), was clearly present in HEK293 cells. However, this was less abundant in the other cell lines and totally absent in the cell extracts from HT29 and HCT116 cells, in which the 65-kDa band was the predominant form. That the smaller band is detected by antibodies to a C-terminal epitope of HECTD3 suggests that it lacks the N-terminal regions of the full-length protein. The observed molecular weight of this smaller species corresponds to that predicted for the translated product of a documented splice-variant mRNA of HECTD3 (NCBI RefSeq XM_011542140.1; predicted molecular weight, 65,687 Da), in which exons 1 and 4 are missing, with translation initiated from a start codon corresponding to Met 285 of the full-length protein. The predicted protein product would start midway through the only part of the N-terminal region of HECTD3 with a recognizable feature—an APC10/DOC1-like domain that, we show, mediates interaction with its CRAF substrate—and would certainly damage the folding and functionality of that putative domain. Consistent with our positive identification of the DOC domain as sufficient for association with CRAF, we found that only the full-length 97-kDa form of HECTD3, but not the 65-kDa N-terminally truncated form lacking an intact DOC domain, was co-immunoprecipitated by EYFP-CRAF from HEK293 cells (Figure 6B).

It is highly likely, therefore, that the shorter isoform found in HCT116 and HT29 cells is not functional in mediating HSP90-directed CRAF degradation in those cells. Consistent with this, while siRNA knockdown of HECTD3 in HCT116 cells (which harbor an activating KRAS mutation) substantially decreased the intensity of the immunoreactive 65-kDa band, it had no effect on the AUY922-triggered degradation of CRAF in those cells (Figure 6C). Taken together, these data identify the 97-kDa isoform with the intact DOC domain as the active form of HECTD3 and suggest that HCT116 cells, which appear to lack the immunoreactive 97-kDa band, also lack functional HECTD3 E3 ubiquitin ligase activity toward CRAF.

## Discussion

Client protein degradation is the mechanism by which inhibitors of the HSP90 chaperone achieve their therapeutic effect, particularly in cancer cells whose growth and/or survival is dependent on HSP90-dependent signaling pathways such as the MAPK cascade (Acquaviva et al., 2014, Garon et al., 2013, Smyth et al., 2014). Whether activated by mutations in KRAS or BRAF, tumorigenic MAPK signaling requires CRAF, which, in turn, depends, for both its cellular stability and activity, on its association with the CDC37-HSP90 molecular chaperone system (Grammatikakis et al., 1999, Pearl, 2005).

As with other HSP90 client protein kinases, impairment of HSP90 function by pharmacological inhibition of its ATPase activity promotes CRAF ubiquitylation and degradation (Eccles et al., 2008, Mimnaugh et al., 1996, Schulte et al., 1995), but the mechanism by which this occurs is poorly understood. In particular, the identity (or identities) of the E3 ubiquitin ligase (or ligases) responsible for specifically recognizing and modifying the client protein substrates is uncertain. Previous studies demonstrated a role for CUL5-based complexes in HSP90-inhibitor-dependent kinase degradation in cancer cells such as HT29 and HCT116 (Ehrlich et al., 2009, Samant et al., 2014) that was surprisingly independent of the TCEB2-TCEB1 (Elongin B-Elongin C) proteins that physically link CUL5 to the SOCS substrate specificity adaptors of that system (Lydeard et al., 2013). We also observed involvement of CUL5, in our screen, in non-cancerous HEK293 cells, but this appears to be more conventional in behavior and dependent on TCEB2-TCEB1.

Here, we have identified HECTD3 as a novel player in the degradation of CRAF, as well as other HSP90 protein kinase clients. HECTD3 is specific for degradation following the inhibition of HSP90’s ATPase activity, but it does not appear to make a major contribution to general CRAF homeostasis or to the chaperone-deprivation pathway triggered by the kinase inhibitor blockade of CDC37 binding (Polier et al., 2013).

Full-length 97-kDa HECTD3 protein was readily detectable in HEK293 cells, but not in tumor cells. HT29 and HCT116 cells express a 65-kDa alternatively spliced isoform, also detectable in HEK293 cells, that is inactive in CRAF degradation. Alteration in mRNA splicing patterns is emerging as a significant mechanism in the progressive acquisition of cancer “hallmarks” by the evolving tumor cell (Oltean and Bates, 2014). Sustaining adequate CRAF protein levels in a tumor cell addicted to MAPK-pathway activation would certainly be favored by adaptive downregulation of a targeted degradation pathway, and alternative splicing of a key E3 ubiquitin ligase to a non-functional isoform, as we observed in the HCT116 cells, would be an effective mechanism to achieve this. HECTD3 may, therefore, have a tumor-suppressive function, controlling the amount of CRAF protein that can be activated through the HSP90 system and thereby limiting MAPK pathway activation.

Relatively little is known about the biochemistry of HECTD3, which has a unique architecture among HECT-domain E3 ubiquitin ligases (Marín, 2010, Rotin and Kumar, 2009) and is poorly characterized at a functional level, although highly conserved in metazoa. HECTD3 has been implicated in the degradation of a handful of proteins, none of which are protein kinases and/or known HSP90 clients (Li et al., 2013a, Li et al., 2013b, Yu et al., 2008, Zhang et al., 2009), but several of these studies utilized overexpressed protein where specificity may be impaired and/or have monitored cellular phenomena that could be downstream of HECTD3’s presumed primary activity as an E3 ligase. We show here the direct involvement of HECTD3 in the degradation of CRAF and other kinases, confirmed by knockdown rather than overexpression.

Our data show that HECTD3 associates strongly with HSP90, CDC37, and CRAF in response to inhibition of the HSP90 ATPase cycle and likely forms a complex with these proteins. Consistent with this, HECTD3 was detected as a potential HSP90 interactor in a large-scale proximity screen (Table S1 in Taipale et al., 2012); interestingly, CUL5 was not detected in that screen. Some HECT-domain E3 ligases recognize their substrates via interaction domains within the same polypeptide chain, while others utilize separate adaptor proteins to mediate substrate interactions in an manner analogous to that of CRL E3 ligases (Rotin and Kumar, 2009). HECTD3 contains a DOC domain structurally related to the APC10 subunit that acts as a degron recognition factor in the APC/C E3 ligase complex (da Fonseca et al., 2011). We show here that this domain, which is disrupted in the alternative spliced inactive isoforms found in CRAF-dependent tumor cell lines, is both necessary and sufficient for HECTD3 to associate with CRAF and with HSP90.

Key to understanding the process of HSP90-mediated degradation of client proteins such as CRAF by HECTD3 and other systems will be the identification of the specific degron recognized by the E3 ligase. Whether this is provided by HSP90, the kinase-specific co-chaperone CDC37, the client kinase itself, or some combination of these, remains to be determined.

## Experimental Procedures

### Expression Plasmid

The gene for the full-length human CRAF was synthesized and cloned into pEYFP-C1 by GenScript as an XhoI-BamHI fragment. Codons were optimized for baculovirus expression.

### Recombinant HECTD3 Expression and Purification

Full-length human HECTD3 was cloned as a BamHI-HindII fragment into pFastbac1 and expressed as a His-tagged fusion in Sf9 cells. The DOC domain (amino acid residues 219–398) and the HECT domain (amino acid residues 512–861) of HECTD3 were cloned as NdeI-HindIII fragments into p3E (Antony Oliver, University of Sussex) and expressed in *E*. *coli*. His_6_-tagged HECTD3 was purified by TALON metal affinity chromatography equilibrated in 50 mM HEPES (pH 7.5), containing 500 mM NaCl. Eluted protein was further purified by size exclusion chromatography using Superdex 200 HR equilibrated in 50 mM Tris, 500 mM NaCl, 1 mM EDTA, and 1 mM DTT (pH 7.5) and Q-Sepharose ion exchange chromatography. GST-DOC and GST-HECT domains were purified using Glutathione Sepharose 4B (GE Healthcare) equilibrated in 20 mM Tris, 140 mM NaCl, 1 mM EDTA, and 1 mM DTT (pH 7.4) and subsequently by Superdex 75 or 200 HR size exclusion chromatography, as appropriate. Both GST-DOC and GST-HECT domain constructs were further purified using Q-Sepharose ion-exchange chromatography.

### In Vitro Binding Assays

HEK293 cells, or HEK293 cells expressing eYFP or eYFP-CRAF, were lysed in 25 mM HEPES (pH 7.8) containing 0.5 mM EDTA, 150 mM NaCl, 10% glycerol, 0.5% Triton-100, and protease inhibitors for 1 hr at 4°C. The cell lysate was clarified by centrifugation at 16,000 × *g* for 25 min at 4°C. Equal amounts of the supernatant (300–500 μL) were then transferred into Eppendorf tubes, and 60 μg His-HECTD3, GST-DOC, and GST-HECT were added into HEK293 cell lysate or cell lysate containing expressed eYFP, as controls, or eYFP-CRAF. The cell lysates were then incubated for 2 hr at 4°C. Meanwhile, GFP-Trap resin (Chromotek, gta-10) was washed three times with 25 mM HEPES (pH 7.8) containing 0.5 mM EDTA, 150 mM NaCl, 10% glycerol, and protease inhibitors and then blocked by incubation with HEK293 cell lysate to reduce subsequent non-specific binding of protein. 30 μL of the pretreated beads were then added to cell lysates, and their ability to co-immunoprecipitate eYFP-CRAF and HECTD3 was analyzed by western blotting.

Essentially, the same procedure was used with cell lysate pretreated for 120 min at 4°C with 0, 100, 200, 400, 800, 1,600 or 3,200 nM AUY922. In these experiments, pretreated GST-Trap resin (Chromotek, sta-200) was used, and co-immunoprecipitations of CRAF and HSP90 were analyzed by western blotting.

### Culture and Cell Line Generation

HCT116 human colorectal carcinoma and HT29 human colorectal adenocarcinoma cell lines were a kind gift from Paul Workman, (The Institute of Cancer Research). Both HCT116 and HT29 cells were grown in DMEM (Life Technologies, 21969-035) supplemented with 10% fetal calf serum (FCS) (homemade), 1 mmol/L non-essential amino acid (Life Technologies, 11140-035), and 1 mmol/L L-glutamine (Life Technologies, 25200-056). HEK293, COS7, U2OS, and A549 cell lines were obtained from the Genome Damage and Stability Centre, University of Sussex. These cell lines were cultured in DMEM supplemented with 10% FCS and 1 mmol/L L-glutamine. All cells were grown at 37°C with a 5% CO_2_ humidified atmosphere.

Stable HEK293 cell lines expressing eYFP-CRAF were generated by transforming cells with peYFP-CRAF using TurboFect (Thermo Fisher, R0531) as the transfection reagent. G418 was used to select stable cell lines and maintained on the standard DMEM described earlier.

### Western Blots and Antibodies

Cells were lysed with 2× NuPAGE LDS Sample Buffer (Life Technologies, NP0007). Cell lysates were then boiled at 100°C for 10–15 min. All samples were then loaded onto SDS-polyacrylamide gels (Life Technologies, NuPAGE 4–12% Bis-Tris Protein Gel, NP0321BOX).

Western blot was carried out on a Bio-Rad Trans-Blot Semi-Dry apparatus using transfer buffer (25 mM Tris [pH 8.5], 192 mM glycine, and 20% methanol) for 50–70 min at 210 V and 120 mA on a nitrocellulose membrane. The membrane was then pre-blocked by incubation in 5% milk powder in PBS (137 mM NaCl, 2.7 mM KCl, 10 mM Na_2_PO_4_, and KH_2_PO_4_ [pH 7.4]) and during the incubation with antibodies. Primary antibodies were incubated with the membrane overnight and washed three times with PBS containing 0.1% Tween 20. The membrane was then incubated with the secondary antibody for 1–2 hr with 2% milk powder in PBS. The primary antibodies used were: α-CRAF (SC-133 and SC-7267), α-BRAF (SC-5284), α-PDK1 (SC-7140), α-CDK4 (SC-601), and α-Ub (SC-8017) from Santa Cruz Biotechnology; α-HECTD3 (ab173122), α-biotin (ab53494), α-LKB1 (ab15095), α-RET (ab134100), and α-HSP90 (ab13492) from Abcam; α-phospho-p44/42 MAPK (9101S), α-HER2/ErbB2 (2242L), α-GFP (2555S), and α-SRC (2108S) from Cell Signaling; α-GAPDH (MA5-15738) from Thermo Fisher; and α-MASTL (A302-190A) from Bethyl Laboratories. Primaries were detected using commercially available HRP (horseradish peroxidase)-conjugated secondary antibodies and visualized either on film or on an ImageQuant LAS500 (GE Healthcare).

### Immunofluorescence Staining

Cells were grown to a 50%–75% confluency on coverslips (Thermo Scientific, A67761333) in six-well plates. These were then washed three times with warm PBS buffer, fixed with 4% paraformaldehyde in PBS for 15 min, and subsequently permeabilized with 0.3% Triton X-100/PBS for 10 min. Following this, cells were blocked for 20 min with BlockAid Blocking Solution (Life Technologies, B10710) and then incubated for 1 hr with the appropriate primary antibody diluted in blocking solution. Slips were subsequently washed and incubated with the correct secondary antibody labeled with a corresponding Alexa Fluor fluorophore. Finally, cells were stained with DAPI prior to imaging.

### Fluorescence Degradation Assay

The fluorescence of eYFP-tagged full-length human CRAF in HEK293 cells was measured on a POLARstar Omega plate reader with an excitation wavelength of 485 nm and an emission wavelength of 520 nm. The fluorescence intensity was recorded at 0, 4, 8, and 12 hr after AUY922 treatment.

### Azide and Alkyne Reaction Pulse Labeling for Newly Synthesized Protein

HEK293 cells stably expressing EYFP-CRAF were transfected with siRNA and grown for 72 hr. All cell samples were then washed twice with warm PBS and cultured in L-methionine-free DMEM (Invitrogen, 21013-024) for 1 hr. Subsequently, the cells were washed twice with warm PBS, before labeling with 30 μM Click-iT AHA (L-azidohomoalanine) (Invitrogen, C10102) for 3 hr, and then washed with warm PBS and regrown in DMEM medium for 24 hr.

Samples were collected without trypsinization, and cell pellets were frozen in liquid nitrogen and stored at −80°C. As required, cell samples were lysed in buffer containing 0.5 mM EDTA, 25 mM HEPES (pH 7.8), 150 mM NaCl, 10% glycerol, 0.5% Triton-100, and 1/100 protease inhibitor for 1 hr at 4°C, and the lysate was clarified by centrifugation at 16,100 × g for 25 min at 4°C. 50 μL of the supernatant (up to 200 μg of protein) containing the AHA-labeled protein sample was used for the AHA-azide and biotin alkyne (Thermo Fisher, B10185) conjugation in the absence of DTT, which is a potent inhibitor of the reaction, using the Click-iT Protein Reaction Buffer Kit (Invitrogen, C10276).

Conjugation was performed by rolling the sample for 20 min at room temperature. Subsequently, the buffer solution of the sample was exchanged on a PD SpinTrap G-25 column (GE, 28-9180-04) equilibrated with 0.5 mM EDTA, 25 mM HEPES (pH 7.8), 150 mM NaCl, 10% glycerol, and 1/100 protease inhibitor. The labeled sample was used in GFP-Trap (Chromotek, gta-10) pull-down experiments. The results were finally analyzed by western blot analysis.

### PLA

Wild-type HEK293 cells were fixed with 4% paraformaldehyde and permeabilized with 0.3% Triton X-100/PBS for 10 min. The ligation experiment was performed according to the manufacturer’s instructions using the Duolink In Situ Red Starter Kit (Sigma-Aldrich, DUO92101). Primary antibodies used were: α-CRAF (SC-133 or SC-7267, Cell Signaling), α-HECTD3 (ab173122, Abcam), and HSP90 (ab13492, Abcam).

### Microscopy and Imaging Processing

All sample images from immunofluorescence assays and PLA were captured by a Leica TCS SP8 confocal microscope. ImageJ or Fiji (for Mac) was used to process the images.

### siRNA Screen

siRNA screening was conducted using the RTF SMARTpool siRNA Library - Human Ubiquitin Conjugation Subset 1 (Dharmacon), consisting of two siRNA-coated plates (H-105615, Lot 12115). Plates were used to reverse-transfect cells at 50 nM final siRNA concentration into optical imaging plates. 72 hr after transfection, eYFP-CRAF degradation was initiated by addition of AUY922 for 8 hr. After incubation, cells were washed and fixed for 10 min using 4% paraformaldehyde in PBS, permeabilized using 0.3% Triton in PBS, and stained with 0.5 μg/mL DAPI in PBS for 15 min. After final wash, all cell samples were immediately imaged using the Olympus ScanR microscope at 10× magnification. Images were subsequently analyzed using ScanR Analysis proprietary software. At minimum 10,000 cells were imaged per individual sample, per experiment.

siRNA verification experiments were conducted in a similar format using specific siRNAs (Table S2). siRNA controls used ON-TARGETplus Non-Targeting Control siRNA #1 (Dharmacon).

### Image Analysis

For analysis, the images were subjected to standardized background correction with a 50-pixel window and image segmentation analysis anchored to imaged nuclei as main objects in order to get the total eYFP-CRAF intensity per nucleus and nucleus-associated cytoplasmic region. To eliminate potential image segmentation artifacts, we included in the analysis only the cells where the associated cell segment was smaller than 9,000 pixels—roughly equivalent to the observed cell size on the raw image. We also excluded high-DNA-content artifacts (>4 N), as they predominantly represented unresolved multi-nucleated cell aggregates. Thus, obtained, filtered data were exported and analyzed using Microsoft Excel. All other immunofluorescence experiments imaged and analyzed on the Olympus ScanR were subjected to the same image-analysis protocol.

### Statistical Analysis

eYFP-CRAF stabilization for experimental siRNA was calculated by the comparison of average fluorescence remaining after treatment with AUY922 for 12 hr with that remaining in AUY922-treated cells transfected with a control siRNA. *Z* scores for each target gene were calculated from the mean and SD of stabilization scores for the full-screen, omitting controls.

## Author Contributions

Conceptualization, L.H.P.; Methodology, Z.L., L.Z., C.P., V.S., and L.H.P.; Investigation, Z.L. and V.S.; Writing – Original Draft, L.H.P.; Writing – Review & Editing, Z.L., C.P., V.S., L.H.P.; Visualization, Z.L. and L.H.P.; Supervision, L.Z., C.P., V.S., and L.H.P.; Funding Acquisition, L.H.P.

## Supplemental Information

Document S1. Figures S1–S3 and Tables S1 and S2

Document S2. Article plus Supplemental Information
