# Supplementary material for: HECTD3 Mediates an HSP90-Dependent Degradation Pathway for Protein Kinase Clients
Source: Cell Rep. 2017 Jun 20;19(12):2515–28. doi: 10.1016/j.celrep.2017.05.078 (PMC5489699; doi:10.1016/j.celrep.2017.05.078)
Supplement: Document S1. Figures S1–S3 and Tables S1 and S2 [file mmc1.pdf]

Cell Reports, Volume *19*

Supplemental Information

HECTD3 Mediates an HSP90-Dependent Degradation Pathway for Protein Kinase Clients

Zhaobo Li, Lihong Zhou, Chrisostomos Prodromou, Velibor Savic, and Laurence H. Pearl

FIGURE S1 Effect of eYFP-CRAF expression on HEK293 cells – related to FIGURE 1

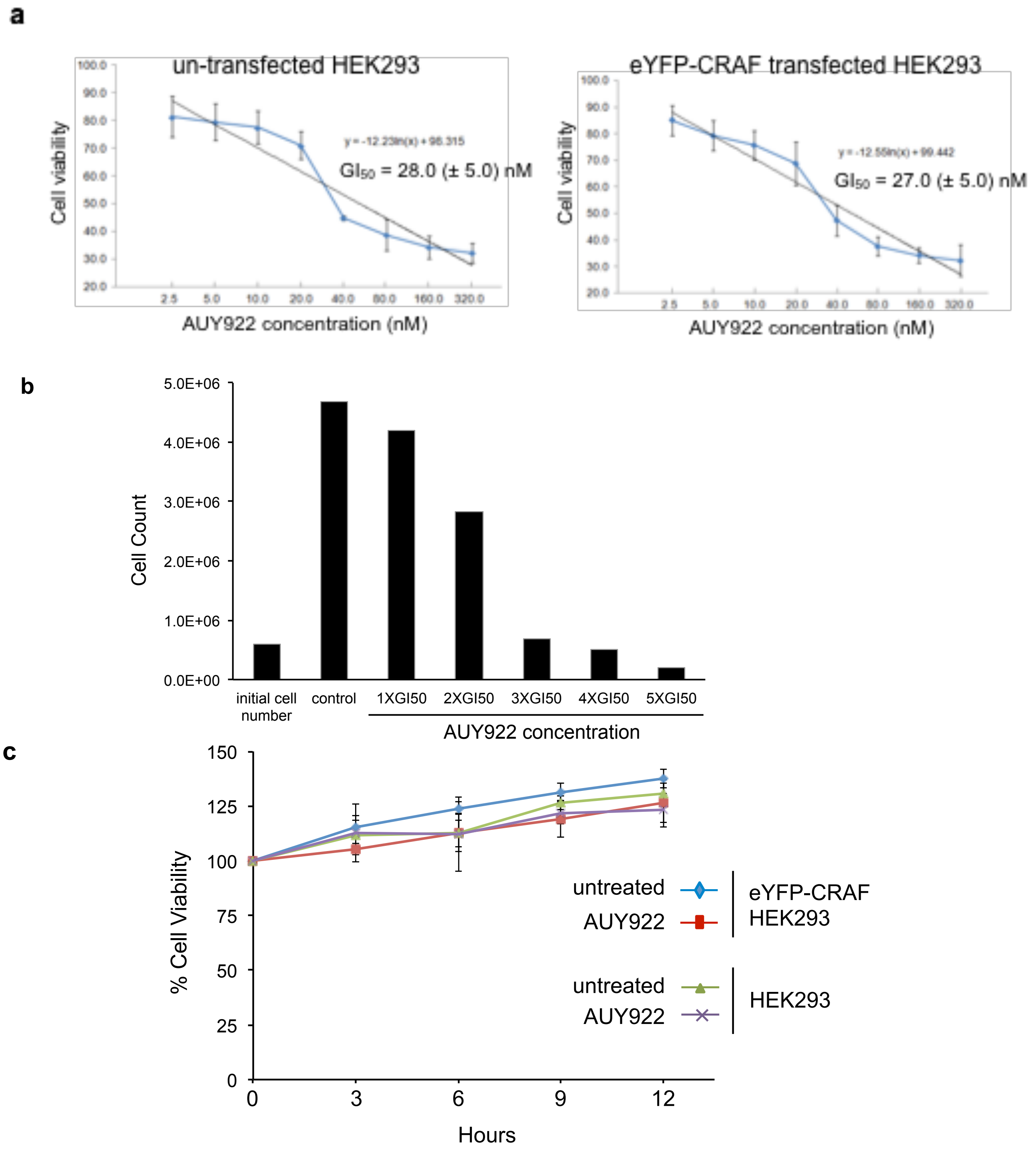

a Determination of GI_{50} - concentration of drug that inhibits cell growth by 50% - for HEK293 cells treated with AUY922 using standard sulphorhodamine B (SRB) assay. Data for un-transfected cells is shown left, that for cells transfected with eYFP-CRAF on right. Expression of eYFP-CRAF has no effect on the susceptibility of the cells to growth inhibition by the HSP90 inhibitor.

b Titration of AUY922 concentration to determine cytostatic dose.

c Expression of eYFP-CRAF and treatment with AUY922 have no substantial effect on cell viability

FIGURE S2 Involvement of HECTD3 in HSP90-independent degradation of CRAF – related to FIGURE 3

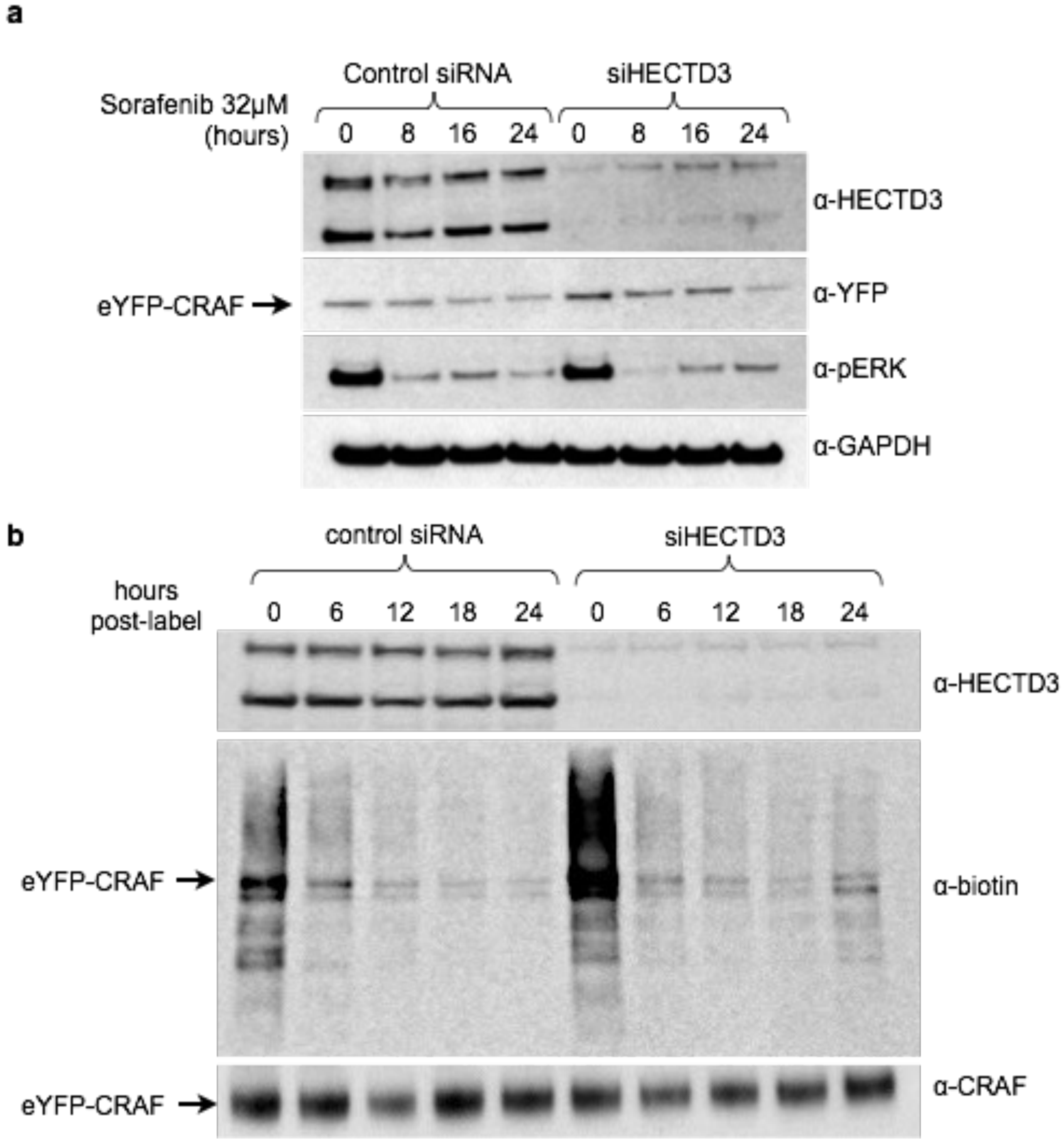

a Western blots showing degradation of eYFP-CRAF in HEK293 cells consequent to chaperone-deprivation by competitive blockade of CDC37 binding to the CRAF kinase domain by the CRAF inhibitor sorafenib. siRNA knock-down of HECTD3 has little effect on eYFP-CRAF degradation by this HSP90-independent degradation pathway compared to siRNA control.

b Western blot of eYFP-CRAF immunoprecipitated from HEK293 cells following pulse-labelling with the methionine mimetic L-azidohomoalanine (AHA) and biotinylated in CLICK reaction. The kinetics of decay of labelled eYFP-CRAF is largely unaffected by siRNA knock-down of HECTD3 compared with control siRNA. Western blot of total eYFP-CRAF provides a loading control.

FIGURE S3 Proximity Ligation Assays – related to FIGURE 4

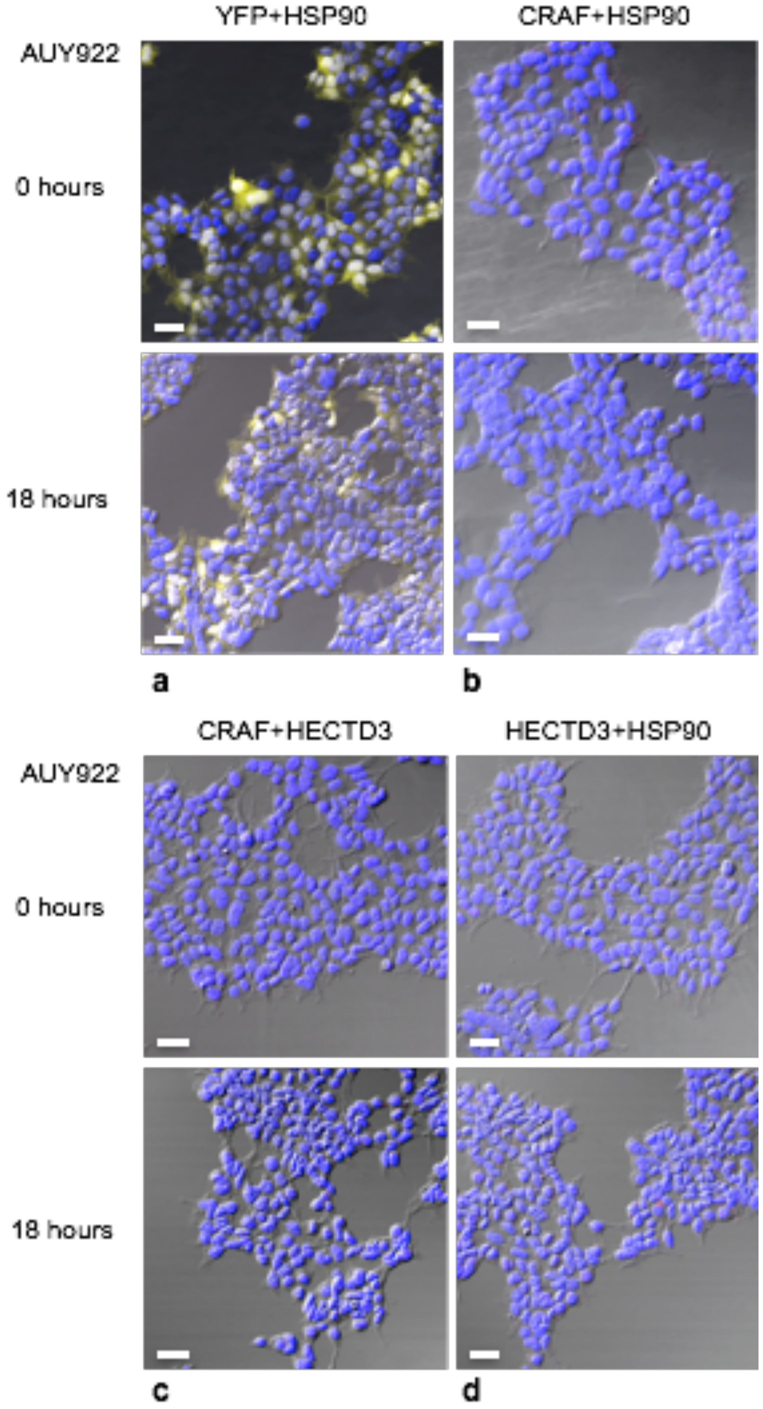

a DIC/Nomarski images of HEK293 cells transfected with eYFP and treated with AUY922 for the times shown. Proximity ligation assay (PLA) generates fluorescent red foci wherever the two target proteins (eYFP and HSP90) come into close proximity. As HSP90 and eYFP are not known interacting proteins, this provides a negative control. Very small numbers of red foci are generated, confirming the specificity of the PLA system. Cell nuclei are stained with DAPI. Scale bar is 25μm. Foci counts are given in **Figure 4b** in the main text.

b As **a** but for CRAF and HSP90. Abundant red foci are visible at 0 hours, and decrease with AUY922 treatment as the CRAF protein becomes channeled into ubiquitylation and degradation.

c As **a** but for CRAF and HECTD3. Small numbers of red foci visible at 0 hours increase with AUY922 treatment as the CRAF protein becomes channeled into ubiquitylation and degradation.

d As **a** but for HECTD3 and HSP90. Foci visible at 0 hours increase with AUY922 treatment as the HECTD3 is recruited into close association with HSP90.

Table S1 – Genes knocked down by siRNA and Z-score of fluorescence stabilization that knock-down produced – related to FIGURE 2

GENE	stabilisation Z-score
HECTD3	4.200950913
UBE1	3.753395451
UBE1DC1	2.733905898
UBE2G1	2.319819517
UBE2D3	1.425621868
CUL5	1.232339666
NEDD4	1.182447771
TSG101	1.10817877
UBE1L2	1.032023824
UBE2E1	1.017409424
DCUN1D4	0.839847552
TRIP12	0.767343153
UBE2I	0.74066953
UBE2J6	0.738242359
UBE2J2	0.69254876
UBE2L3	0.636417808
UBE2Z	0.533085282
ARIH1	0.478533594
UBE2Q1	0.436921992
WWP2	0.433868291
UEVLD	0.375980471
HERC1	0.36775933
HERC3	0.323390523
HERC4	0.315556526
UBE2H	0.21374207
UBE2W	0.187565152
UBE2M	0.182202262
UBE2V2	0.166587932
HECTD2	0.148282269
CUL7	0.144586167
UBE3A	0.129687186
UBE1L	0.104961405
HECW1	0.074230698
UBE2D2	-0.036490817
UBE2J1	-0.056565264
HERC6	-0.088625276
AKTIP	-0.088802237
UBE1C	-0.11598847
WWP1	-0.134338081
UBE2F	-0.146398379
UBE2U	-0.160265933
UBE2O	-0.178178807

CUL2	-0.184433852
UBE2D4	-0.186682487
DCUN1D2	-0.193359428
UBE2B	-0.202951413
UBE2NL	-0.219468371
DCUN1D1	-0.221589014
CUL4B	-0.2266379
NEDD4L	-0.23521394
UBE2Q2	-0.244762594
CUL3	-0.251760911
HACE1	-0.31084695
UBE2S	-0.35217117
CUL4A	-0.364237196
ITCH	-0.364636888
CAND2	-0.389070017
SMURF1	-0.420583542
HECW2	-0.43609391
BIRC6	-0.488965306
UBE3B	-0.512491063
HECTD1	-0.520819141
UBE2V1	-0.539011695
UBE2C	-0.585413952
HUWE1	-0.594849647
HIP2	-0.59499696
HERC5	-0.632480073
UBE2N	-0.63949251
FLJ34154	-0.666199727
UBE2E3	-0.702214336
SMURF2	-0.732166025
UBE2D1	-0.752771829
FLJ25076	-0.77004678
DCUN1D3	-0.78126792
UBE2R2	-0.859697353
HERC2	-0.881168846
UBE2G2	-0.92428632
UBE2A	-0.961184861
UBE2E2	-1.00244961
DCUN1D5	-1.003312091
C10ORF46	-1.054945839
CDC34	-1.056789233
KAA0317	-1.125955123
UBE2T	-1.15319266
TIP120A	-1.343741965
CUL1	-1.626189109
UBE3C	-2.184078934

Table S2 – siRNAs used for verification of screen hits – related to FIGURE 2

Commercial :

ON-TARGETplus Human UBE2E1- Individual	J-008850-07-0005
ON-TARGETplus Human UBA1- Individual	J-004509-05-0002
ON-TARGETplus Human TCEB1- Individual	J-010541-09-0002
ON-TARGETplus Human TCEB2- Individual	J-012376-08-0002
ON-TARGETplus Human STIP1- Individual	J-019802-05-0020
ON-TARGETplus Human UBE2G1- Individual	J-010154-06-0005
ON-TARGETplus Human UBE2D3- Individual	J-008478-10-0005
ON-TARGETplus Human NEDD4- Individual	J-007178-08-0005
ON-TARGETplus Human NEDD4- Individual	J-007178-06-0005
ON-TARGETplus Human STUB1- Individual	J-007201-07-0002
ON-TARGETplus Human UBA5- Individual	J-006405-05-0002
ON-TARGETplus Human UBA6- Individual	J-006403-09-0002

Custom siRNA :

CUL5-01: CAGCTGGTTATTGGAGTAAGA

CUL5-02: CTGGAGGACTTGATACCGGAA

CUL5-03: CAGGTTTGAATCAGTCACCTA

CUL5-04: CCAGCTGATTCAGTTATTATA

HECTD3-01: GCGGGAACUAGGGUUGAAUUU

HECTD3-02: GGUAUUUCACCUCUUAAGAUU
